# Beclin 1 acetylation impairs the anticancer effect of aspirin in colorectal cancer cells

**DOI:** 10.18632/oncotarget.20367

**Published:** 2017-08-19

**Authors:** Ting Sun, Liang Ming, Yunmeng Yan, Yan Zhang, Haikuo Xue

**Affiliations:** ^1^ Key Clinical Laboratory of Henan Province, Department of Clinical Laboratory, The First Affiliated Hospital of Zhengzhou University, Zhengzhou 450052, China; ^2^ Department of Medical Laboratory, Zhengzhou University, Zhengzhou 450001, China

**Keywords:** aspirin, autophagy, Beclin 1, acetylation, colorectal cancer

## Abstract

Regular use of aspirin can reduce cancer incidence, recurrence, metastasis and cancer-related mortality. Aspirin suppresses proliferation and induces apoptosis and autophagy in colorectal cancer cells, but the precise mechanism is not clear. In this study, we demonstrated that aspirin induced autophagosome formation in colorectal cancer cells, but autophagic degradation was blocked through aspirin-mediated Beclin 1 acetylation. Blocked autophagic degradation weakened aspirin-induced cell death. Collectively, our findings indicate the dual roles of aspirin on autophagy, and demonstrate a new mechanism by which Beclin 1 acetylation impairs the anticancer effect of aspirin in colorectal cancer cells.

## INTRODUCTION

Aspirin, a nonsteroidal anti-inflammatory drug (NSAID), is widely used as a painkiller, antipyretic or antiplatelet agent for more than 100 years [1]. In addition to its classical anti-inflammatory function, epidemiological studies in several trials have demonstrated that prolonged aspirin use reduces cancer risk, particularly colorectal cancer (CRC) [2–7], indicating a promising role of aspirin for cancer prevention [8–10]. Although evidence of aspirin’s anticancer effect is compelling, the underlying molecular mechanism remains enigmatic.

Aspirin consists of acetyl and salicylate moieties. While the salicylate group implicates in the anti-inflammatory and anti-cancer properties via targeting cyclin A2/CDK2, HMGB1 and NF-κB pathway [11–13], the acetyl group causes the inactivation of cyclooxygenases (COXs) through acetylation of serine residues [14]. While aspirin's ability to acetylate and inhibit COXs enzyme activity is well known [14], multiple cellular proteins can be acetylated by aspirin, suggesting that aspirin may exert its anticancer effect by acetylating multiple cellular targets [15, 16].

Substantial evidence indicates aspirin induces apoptosis and autophagy [17–19]. Autophagy is a highly conserved self-digestion process, during which useless cytoplasmic components, such as protein aggregates, damaged organelles, are sequestered into double-membraned structures called autophagosomes. Autophagosomes then fuse with lysosomes for subsequent degradation [20–22]. Beclin 1 is an essential autophagy effector. Our previous study has confirmed that Beclin 1 acetylation inhibits autophagosome maturation and promotes tumor growth [23].

In this study, we investigated the effect of aspirin on Beclin 1 acetylation and autophagy in CRC cells, providing new insight into aspirin for cancer therapy.

## RESULTS

### Aspirin induces autophagosome formation in colorectal cancer cells

**Figure 1:**
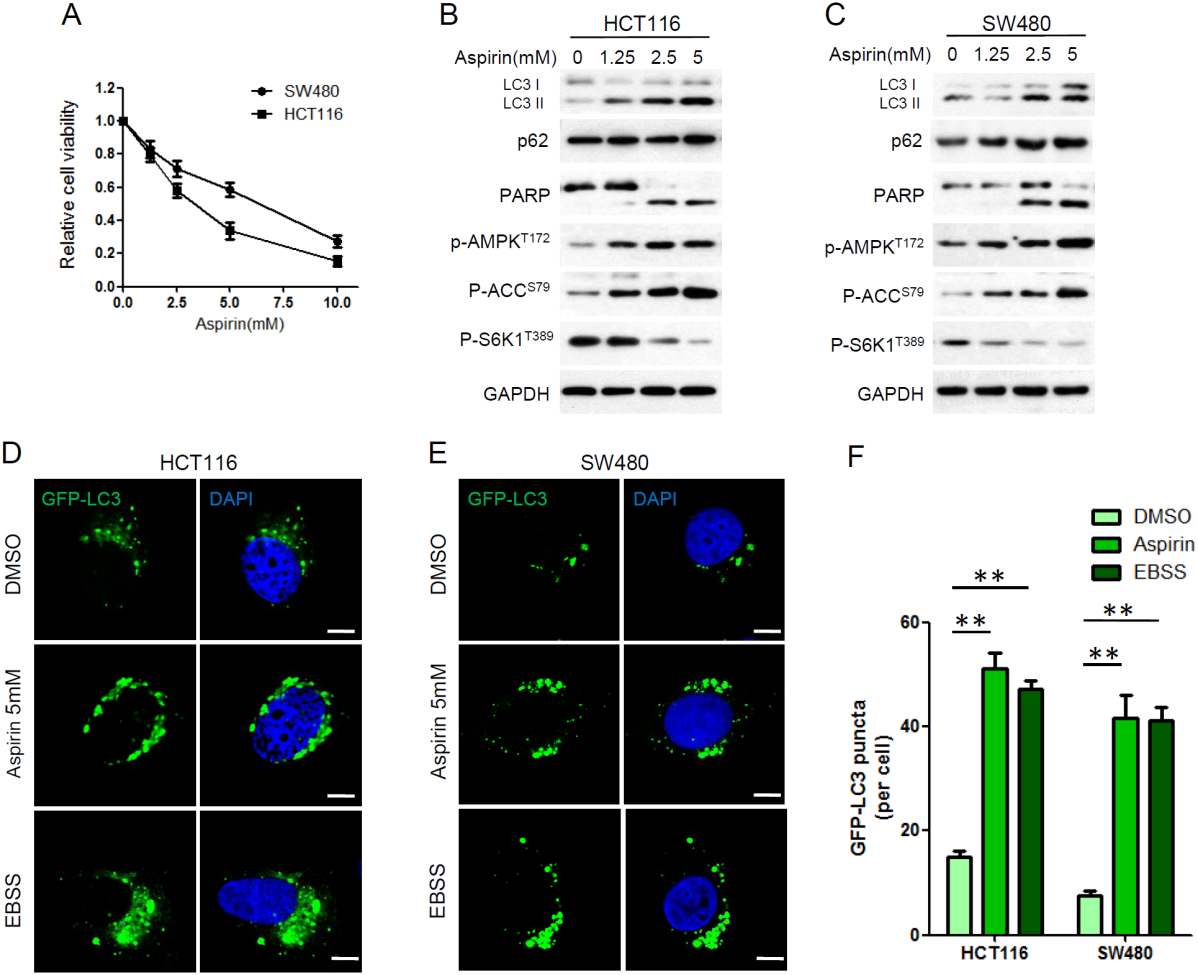
Aspirin inhibited proliferation and induced autophagy in HCT116 and SW480 cells **(A)** Cell viability was determined by CCK8 assay after aspirin treatment at various concentrations (0, 1.25, 2.5, 5, 10 mM) for 72 h. Bars represent mean ± SEM. Three independent experiments (n=3). **(B-C)** HCT116 and SW480 cells were treated with different concentration of aspirin for 24 h. Protein levels were estimated using western blot analysis. GAPDH was used as loading control. **(D-E)** HCT116 and SW480 cells were transfected with GFP-LC3 plasmid for 24 h. Then the cells were treated with 5mM aspirin or DMSO for 24h or EBSS for 4h, followed by confocal fluorescence microscopy. Scale bar, 5μm. Data represents three independent experiments. **(F)** Quantitation of GFP-LC3 puncta in D and E. Bar are mean ± SEM of 50 cells; three independent experiments, **P< 0.01 (Student’s t-test).

Substantial evidence indicates that aspirin inhibits cell proliferation. A recent publication shows that aspirin inhibits cell proliferation through downregulation of c-myc gene expression in HCT116 cells [24]. In our study, the antiproliferative activity of aspirin on CRC cells was investigated by CCK-8 assay. Treatment of HCT116 and SW480 cells with varying concentrations of aspirin resulted in significantly reduced survival of cells in a concentration-dependent manner (Figure 1A). These results confirmed the potential cytotoxic effect of aspirin in CRC cells.

Previous study indicates aspirin could induce apoptosis [25, 26]. We assessed the effect of aspirin on the expression of apoptosis marker PARP. Cleavage of PARP was observed in HCT116 and SW480 cells after aspirin treatment, confirming induction of apoptosis by aspirin (Figure 1B, 1C). Previous study has proved that aspirin inhibits mTOR, activates AMPK, and induces autophagy in colorectal cancer cells [19]. We also confirmed this in our study. LC3 is a commonly used autophagy marker and its processed form, LC3 I, resides in cytoplasm. After autophagy induction, LC3 II, the conjugated form of LC3, associates with autophagosomes. Substantial up-regulation of LC3 II was observed in HCT116 and SW480 cells after aspirin treatment (Figure 1B, 1C). We also investigated aspirin’s effects on the activity of mTORC1 and AMPK. ACC (acetyl-CoA carboxylase) is one direct downstream target of AMPK. There was a striking decrease in the mTORC1 target protein S6K phosphorylation, while phosphorylation of AMPK and ACC was increased after aspirin treatment, confirming aspirin induces AMPK activation and mTOR inhibition in CRC cells (Figure 1B, 1C).

Aspirin-induced autophagy induction was further confirmed by immunofluorescence. HCT116 and SW480 cells were transfected with GFP-LC3, a highly specific fluorescent marker of autophagy, to measure autophagosome formation. We also use Earle’s balanced salt solution (EBSS) to mimic the nutrient-starvation condition to induce autophagy. Aspirin increased GFP-LC3 puncta significantly, just as EBSS did, confirming autophagosome formation was induced by aspirin in CRC cells (Figure 1D-1F).

### Aspirin inhibits autolysosome degradation in colorectal cancer cells

Autophagy is a highly dynamic, multi-step process, including autophagosome formation, maturation, fusion with lysosomes and degradation [27]. Therefore, an increase in autophagosomes alone, does not necessarily indicate increased autophagy flux [28]. p62 is a polyubiquitin-binding protein which contains a LC3-interacting motif and an ubiquitin-binding domain. By linking ubiquitinated substrate with autophagic machinery, p62 is incorporated in completed autophagosomes and degraded in autolysosomes, together with its bound proteins [29]. As shown in Figure 1B and 1C, p62 was not degraded in aspirin-induced autophagy. It suggested that autophagy-mediated clearance of p62 was blocked.

**Figure 2:**
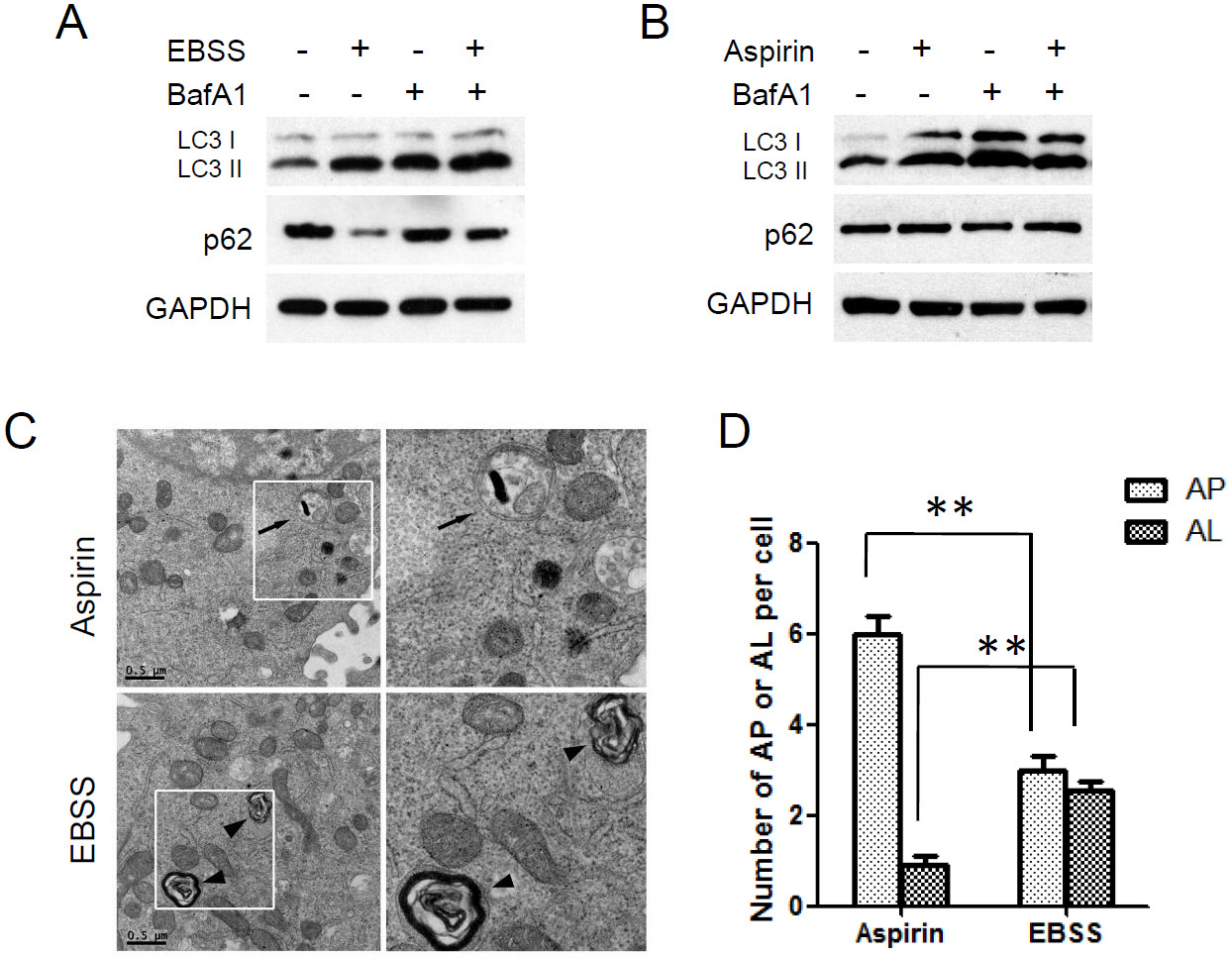
Autophagic degradation was blocked in aspirin treated SW480 cells **(A)** HCT116 cells were treated with EBSS for 4 h after cells were pretreated with 100 nM Bafilomycin A1 for 2 h. **(B)** HCT116 cells were treated with aspirin 5mM for 24 h after cells were pretreated with 100 nM BafilomycinA1 for 2 h. Levels of LC3 and p62 were evaluated by western blotting. GAPDH was used as loading control. **(C)** Electron microscopy analysis of autophagosomes and autolysosomes. HCT116 cells treated with EBSS or Aspirin were subjected to electron microscopy analysis. The arrows indicate autophagosomes (AP) and the arrowheads indicate autolysosomes (AL). Scale bar, 0.5μm. **(D)** Quantitation of AP and AL in HCT116 cells as in C. Bar are mean ± SEM of 20 cells; three independent experiments, **P< 0.01 (Student’s t-test).

Bafilomycin A1 (BafA1), a vacuolar H+-ATPase inhibitor, inhibits the degradation of autophagosomes by preventing lysosome function [30]. EBSS induced the conversion of LC3 I to LC3 II and downregulation of p62 in HCT116 cells, and the accumulation of LC3 II was further enhanced by bafilomycin A1. Bafilomycin A1 also restored the downregulation of p62 (Figure 2A). Therefore EBSS induces a true increase in autophagic flux. However, things were different in aspirin induced-autophagy. Aspirin induced the accumulation of LC3 II but not the downregulation of p62 in HCT116 cells. And bafilomycin A1 nearly had no effect on the aspirin-induced accumulation of LC3 II (Figure 2B).

We also examined the intracellular morphologic change of HCT116 cells by using transmission electron microscopy. Electron microscopy analysis showed that many autophagosomes accumulated in aspirin-treated cells, while autolysosomes were relatively rare. But in EBSS-treated cells, autophagosomes and autolysosomes were observed simultaneously (Figure 2C, 2D). In conclusion, these findings indicated that aspirin induces autophagosome formation but these autophagosomes can not fusion with lysosomes and degradation.

### Aspirin promotes acetylation of Beclin 1

**Figure 3:**
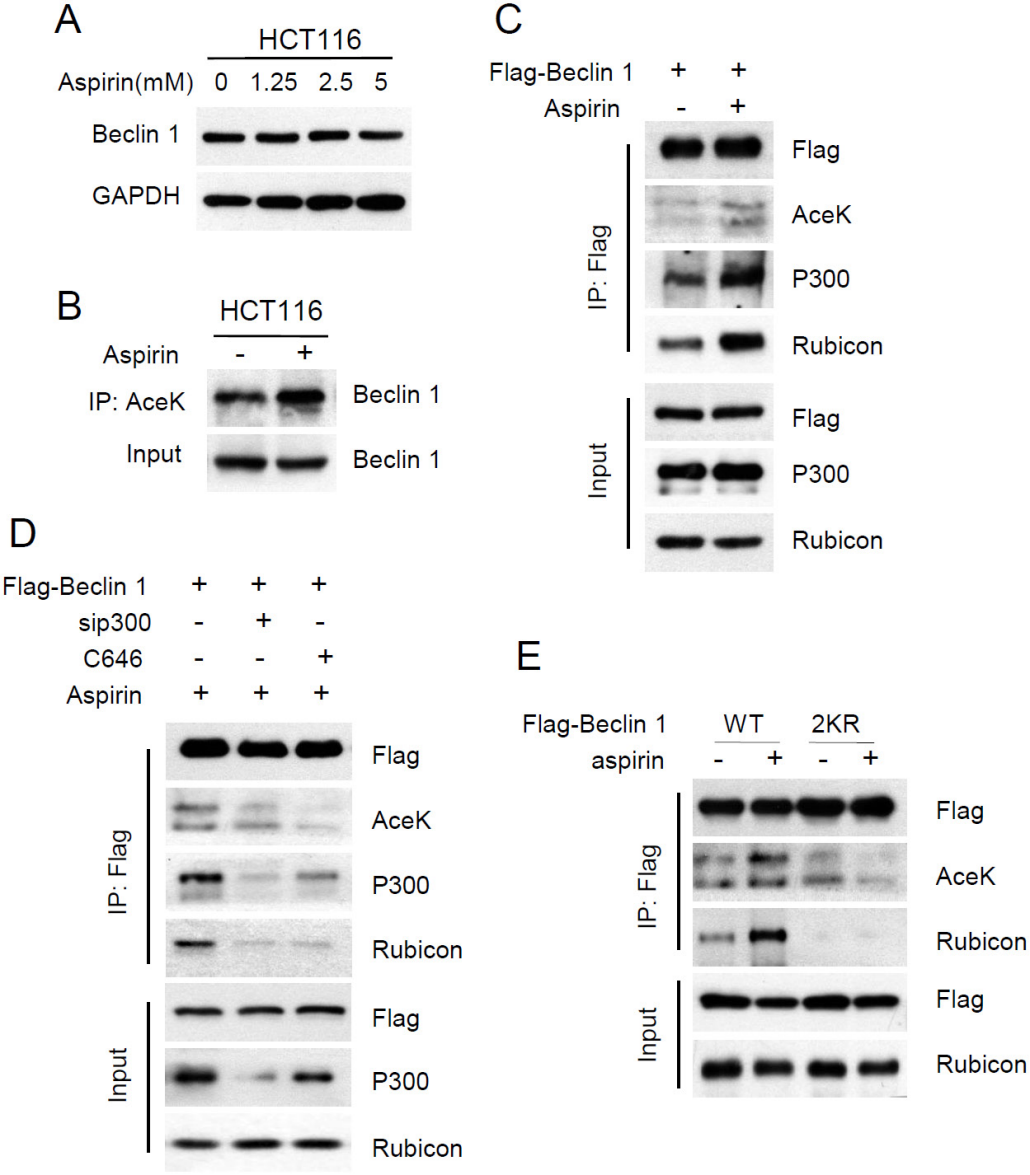
Aspirin enhanced p300-mediated Beclin 1 acetylation on lysine 430 and 437 **(A)** HCT116 cells were treated with different concentration of aspirin for 24 h. Protein levels of Beclin 1 were estimated using western blot analysis. GAPDH was used as loading control. **(B)** Acetylated proteins were immunoprecipitated with the antibody to acetylated lysine from HCT116 cells after aspirin treatment. Acetylation of endogenous Beclin 1 protein was analyzed with western bolt. **(C)** Flag-tagged Beclin 1 was transfected into HCT116 cells. Then the transfected cells were treated with DMSO or 5mM aspirin for 24 h. Acetylation of exogenous Beclin 1 protein was analysed with IP and western bolt. **(D)** Flag-tagged Beclin 1 was transfected into HCT116 cells, in another group, flag-tagged Beclin 1 was co-transfected with sip300. Then the transfected cells were treated with aspirin DMSO or C646. **(E)** Flag-tagged Beclin 1 (WT, 2KR) was transfected into HCT116 cells, then the transfected cells were treated with DMSO or 30μM C646 for 24 h. Beclin 1 acetylation and the interaction between Beclin 1 and p300 or Rubicon was determined with IP and western blot analyses.

Our previous study has proved that Beclin 1 can be acetylated by acetyltransferase p300, and Beclin 1 acetylation inhibits autophagosome maturation and degradation by promoting the binding of Rubicon to Beclin 1 [23]. Evidence also shows that aspirin promotes p300-mediated acetylation of COMMD1 [31]. So we investigated whether aspirin affects the acetylation of Beclin 1 to inhibit autophagic degradation. Firstly, we examined the effect of aspirin on protein expression of Beclin 1. The protein levels of Beclin 1 did not change with aspirin treatment in HCT116 cells (Figure 3A). Then the acetylation level of endogenous Beclin 1 was detected by using an anti-acetylated lysine antibody. We found that acetylation of Beclin 1 was significantly increased after treating cells with aspirin (Figure 3B). Moreover, HCT116 cells were transiently transfected with flag-tagged Beclin 1. Similar experiments with ectopically expressed Beclin 1 also showed that aspirin enhances Beclin 1 acetylation and the binding of Beclin 1 to p300 and Rubicon (Figure 3C). To further validate our finding, we knocked down p300 by target siRNA or blocked p300 activity with inhibitor C646. p300 knockdown decreased Beclin 1 acetylation and the interaction with Rubicon induced by aspirin. Similar effects were observed when p300 was inhibited by 30μM C646 (Figure 3D).

Our previous study has confirmed that Beclin 1 is acetylated by p300 at lysine 430 and lysine 437 [23]. The acetylation sites on Beclin 1 were first identified by mass spectrometry and site-directed mutagenesis of Beclin 1 was performed. We mutated each or both lysine (K) to arginine (R), each single mutation resulted in a weak reduction in Beclin 1 acetylation, whereas the double mutation (2KR) resulted in a significant decrease in Beclin 1 acetylation, indicating that Beclin 1 can be acetylated at the site Lys430 and Lys437. In this study, we investigated whether aspirin-mediates Beclin 1 acetylation was on the same amino acid residues. Beclin 1 lysines 430 and 437 were mutated to arginine (2KR). We then transfected wildtype or 2KR mutant Beclin 1 into HCT116 cells. Aspirin increased the acetylation of wild-type but not 2KR mutant Beclin 1. Correspondingly, aspirin also resulted in the enhanced interaction of Rubicon with WT but not with the 2KR mutant Beclin 1 (Figure 3E). These findings indicate that aspirin promotes p300-mediated Beclin 1 acetylation at lysines 430 and 437 in CRC cells.

### Aspirin-mediated Beclin 1 acetylation inhibits autophagic degradation

**Figure 4:**
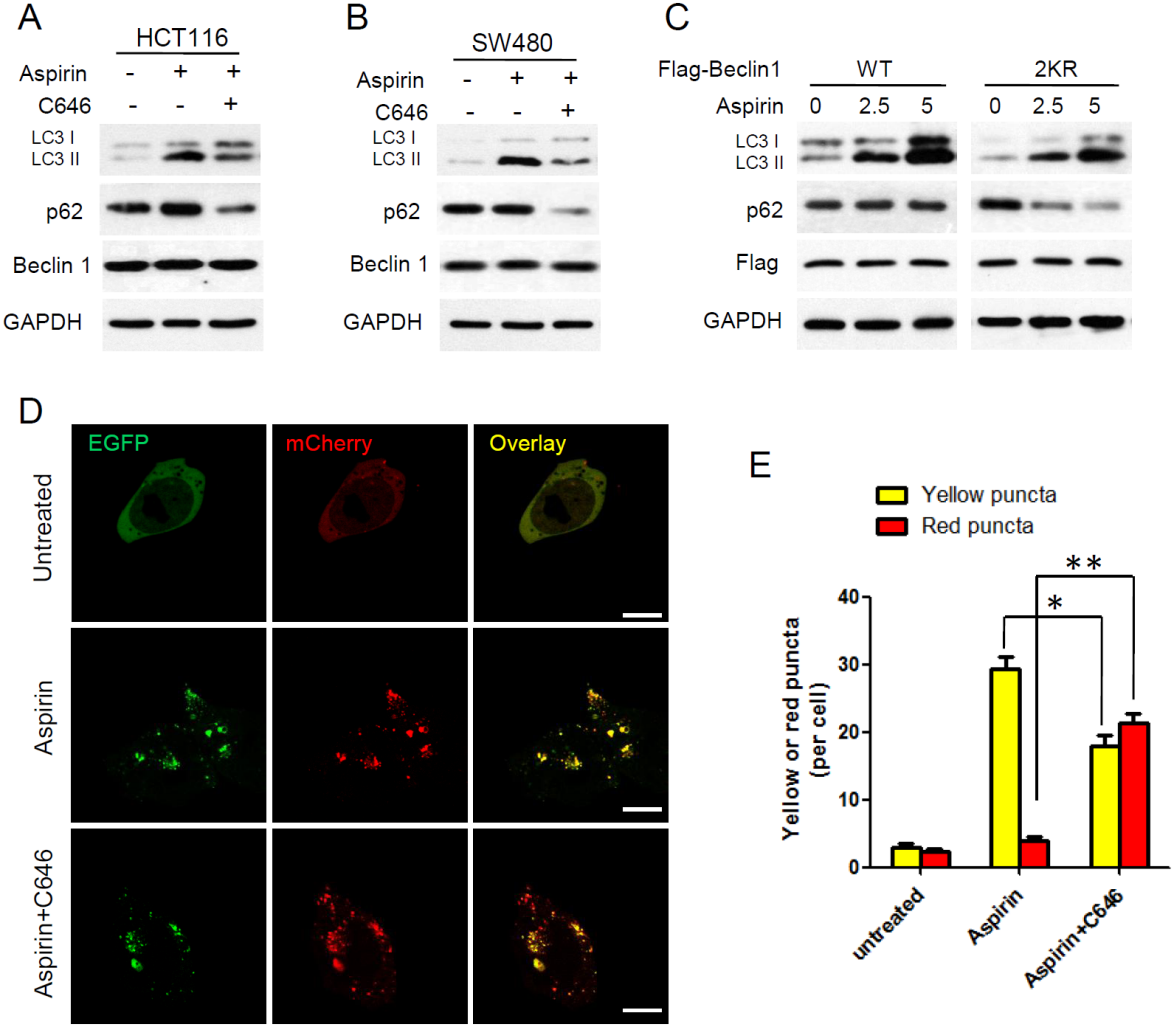
Aspirin-mediated Beclin 1 acetylation inhibits autophagic degradation **(A-B)** HCT116 and SW480 cells were treated with or without 30μM C646 in the presence or absence of 5mM aspirin for 24 h, and western blot analyse of LC3, p62 and Beclin 1 were performed. **(C)** Flag-tagged Beclin 1 (WT, 2KR) was transfected into HCT116 cells, then the transfected cells were treated with different concentration of aspirin for 24 h. Western blot analyses of LC3 and p62 were performed. **(D)** HCT116 cells transfected with mCherry-EGFP-LC3 were treated with or without 30μM C646 in the presence of 5mM aspirin for 24 h. Scale bars, 5μm. **(E)** Quantitation of red and yellow puncta in D. Bars represent mean ± SEM of 50 cells; three independent experiments, *P<0.05, **P<0.01 (Student’s t-test).

Aspirin-mediated Beclin 1 acetylation enhanced the binding of Beclin 1 with Rubicon, which is a negative regulator for autophagosome maturation and degradation [32, 33]. To further assess the effect of Beclin 1 acetylation on aspirin induced autophagy, we performed western blot analysis of p62 and LC3. HCT116 and SW480 cells were treated with aspirin or together with p300 inhibitor C646. We found that combination with C646 released the block of p62 degradation induced by aspirin and the accumulation of LC3 II was also attenuated slightly. However, Beclin 1 protein expression did not change in various treatments (Figure 4A, 4B). We also assess the different effect of aspirin on autophagic degradation in wildtype or the 2KR mutant Beclin 1 expressing HCT116 cells. After aspirin treatment, p62 was not degraded in cells expressing Beclin 1 WT, but degradation of p62 was accelerated in the Beclin 1-2KR expressing cells. And the accumulation of LC3 II was higher in WT Beclin 1 expressing cells (Figure 4C). These findings indicated that aspirin-mediated Beclin 1 acetylation inhibits p62 degradation in lysosomes.

Furthermore, tandem fluorescent mCherry-EGFP-LC3 was designed to monitor autophagic flux. The EGFP signal is acid sensitive and quenched quickly following fusion with lysosomes, whereas mCherry is relatively stable. Therefore, colocalization of both EGFP and mCherry fluorescence indicates an early autophagosome (yellow) and an mCherry signal only corresponds to an autolysosome (red) [34]. Before treatment with aspirin, only weak signals of EGFP and mCherry which represent diffuse LC3 protein were found in the cytoplasm. After treatment with aspirin, a large number of yellow puncta was observed in the perinuclear region, suggesting the formation of autophagosomes, but red puncta were relatively rare. When combination C646 with aspirin, more red puncta were observed than aspirin treated only (Figure 4D, 4E). It suggested that C646 promotes autophagosomes develop maturing to autolysosome in aspirin treated cells.

Taken together these findings indicated that degradation is blocked by Beclin 1 acetylation in aspirin-induced autophagy, and p300 inhibitor releases the block of autophagic degradation.

### Inhibition of Beclin 1 acetylation enhances aspirin cytotoxic effect in CRC cells

**Figure 5:**
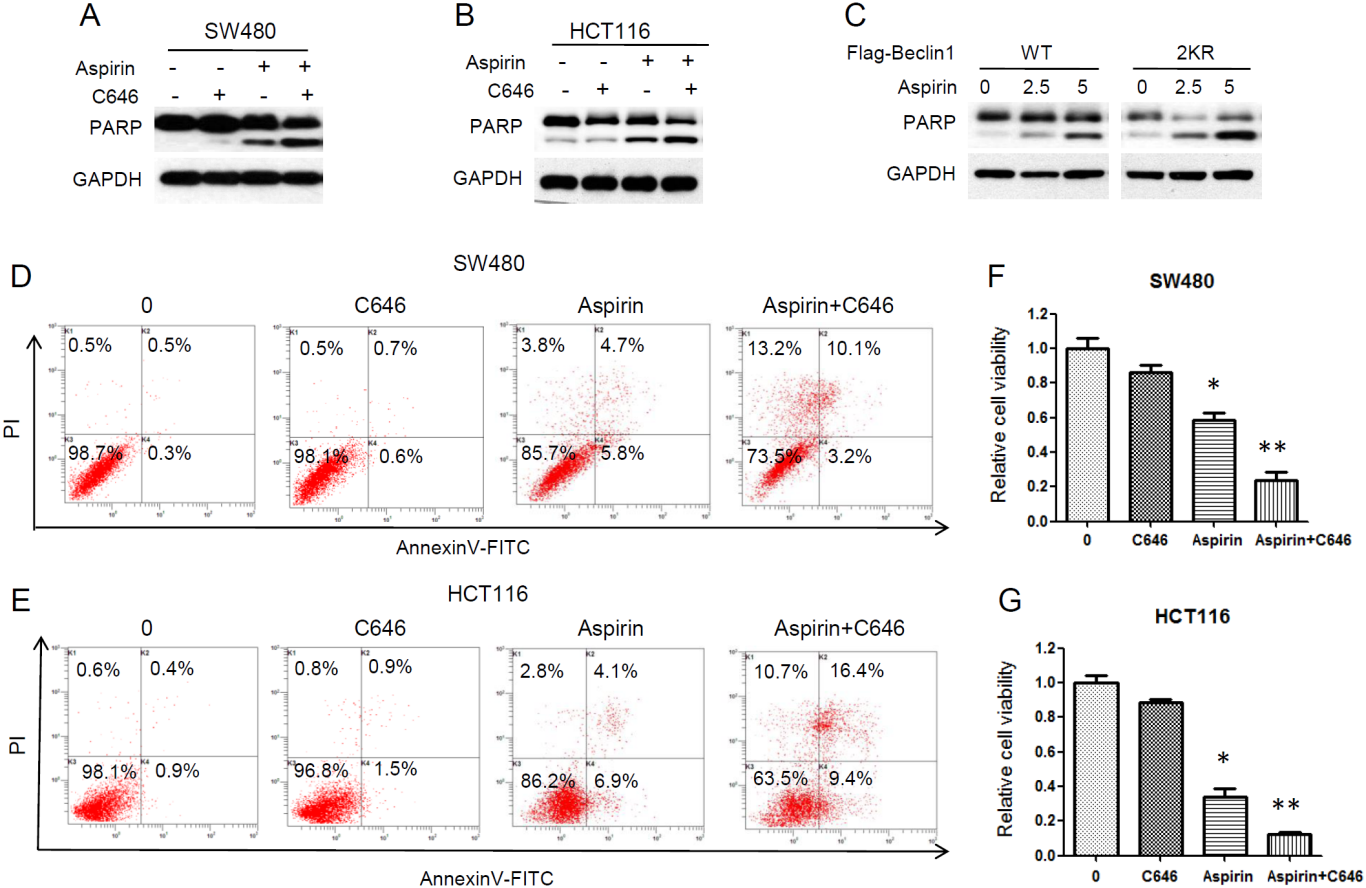
Inhibition of Beclin 1 acetylation by C646 promoted aspirin-induced cell death **(A-B)** HCT116 and SW480 cells were treated with or without 30μM C646 in the presence or absence of 5mM aspirin for 24 h, and western blot analyse of PARP was performed. **(C)** Flag-tagged Beclin 1 (WT, 2KR) was transfected into HCT116 cells, then the transfected cells were treated with different concentration of aspirin for 24 h. Western blot analyse of PARP was performed. **(D-E)** Flow cytometric assay with Annexin V-FITC/PI double staining. HCT116 and SW480 cells were treated with or without 30μM C646 in the presence of 5mM aspirin for 24 h, stained with AnnexinV-FITC and PI, then measured by flow cytometer. **(F-G)** HCT116 and SW480 cells were treated with 5mM aspirin with or without 30μM C646 for 72 h. Cell viability was then determined using CCK-8 assays. Bars represent mean ± SEM. Three independent experiments. *P<0.05, **P<0.01 (Student’s t-test).

To further determine the effect of Beclin 1 acetylation on aspirin-induced apoptosis, we inhibited Beclin 1 acetylation by C646 in aspirin treated CRC cells. Combination with C646 resulted in more cleavage of PARP than aspirin only in HCT116 and SW480 cells (Figure 5A, 5B). Aspirin also induced more cleavage of PARP in the Beclin 1-2KR expressing cells than cells expressing wide-type Beclin 1 (Figure 5C). Annexin V/PI apoptosis assay was performed by flow cytometer. Combination C646 with aspirin resulted in obviously more apoptosis in HCT116 and SW480 cells than aspirin treatment only and C646 itself nearly induced no apoptosis in CRC cells (Figure 5D, 5E). It is worth noting that combination C646 with aspirin resulted in some non-apoptotic cell death (Annexin V-/PI+). These findings suggested that, besides apoptosis, autophagy also takes part in the aspirin-mediated cell death in CRC cells.

We next evaluated the role of Beclin 1 acetylation on antitumor efficacy of aspirin. We found that inhibiting Beclin 1 acetylation by C646 potentiated the cytotoxic effect of aspirin in HCT116 and SW480 cells (Figure 5F, 5G). These results suggested that Beclin 1 acetylation acts as a pro-survival mechanism in aspirin induced cell death and unrestricted autophagic degradation enhances the antitumor efficacy of aspirin in CRC cells.

## DISCUSSION

Considerable interest has emerged over the last decade regarding the role of aspirin in prevention of CRC [35–37]. Besides inhibition of COX, aspirin has a diversity of tumor suppressive effects through COX-independent mechanisms [38–40]. Despite a lot of epidemiological and preclinical studies that test the tumor suppressive role of aspirin, little is known about the roadblocks that counteract the anticancer effect of aspirin.

Studies over the past decades suggest that aspirin acetylates many cellular proteins [15, 16]. Since post-translational modification of proteins, such as acetylation, may lead to the alteration of their function, it is possible that some of the hitherto unexplained therapeutic properties of aspirin may occur as a result of these modifications. The identification of these novel acetylation targets of aspirin represents a new area for investigation. In this study, we demonstrate for the first time that aspirin acetylated Beclin 1 in HCT116 and SW480 colorectal cancer cells. Thus, our finding extends the previously known list of proteins acetylated by aspirin suggesting that the acetylation of proteins may be a major factor involved in some of the unexplained effects of aspirin.

**Figure 6:**
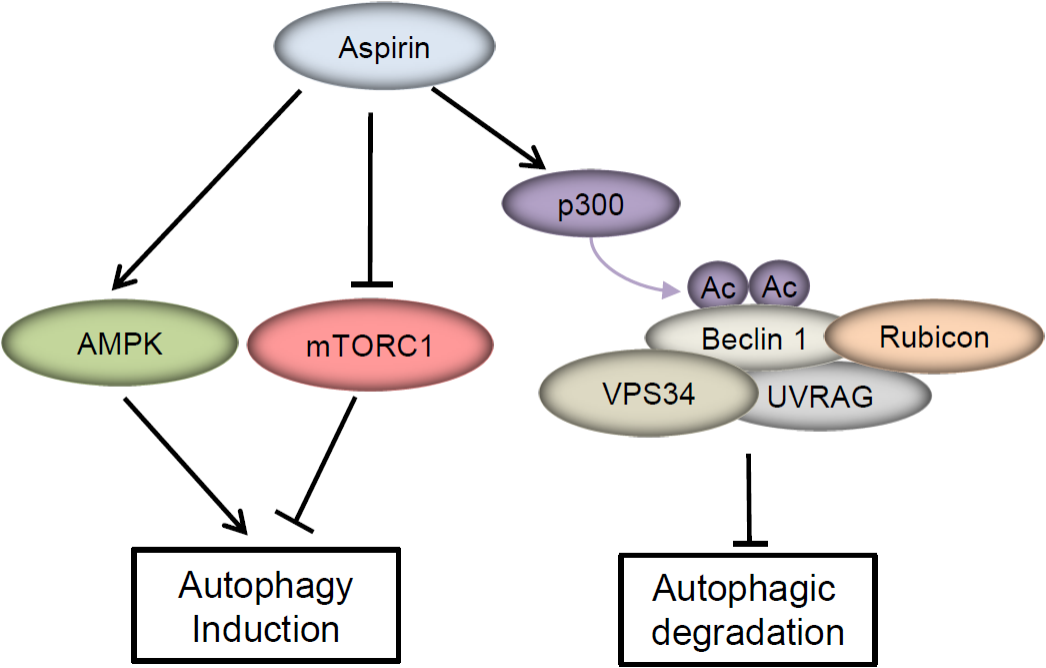
A model of the molecular linkage between aspirin, Beclin 1 acetylation and autophagy

The ability of aspirin to trigger apoptosis in cancer cells is well known. Besides apoptosis, autophagy is frequently reported to be induced by many antitumor agents. Farhat V.N. Din et al reported that aspirin induces autophagy in colorectal cancer cells through AMPK activation and mTOR inhibition. In this study, we found that aspirin induces autophagosomes formation, but the autophagosome induced by aspirin can not go through to degradation in lysosomes. Aspirin-mediated Beclin 1 acetylation inhibited autophagosome degradation, then weakened the aspirin-induced cell death in CRC cells (Figure 6). On the contrary, the complete autophagic flux enhanced the cytotoxicity of aspirin in CRC cells. These findings indicate that Beclin 1 acetylation serves as the roadblock that counteract the anticancer effect of aspirin.

Aspirin is also known as acetylsalicylic acid (ASA), with salicylic acid serving as the important active metabolite. Our study shows that aspirin-mediated Beclin 1 acetylation impairs the anticancer effect of aspirin in colorectal cancer cells. In order to clarify whether the observed effects are due to acetylation effect of aspirin rather than function of salicylate group. We confirmed autophagy occurrence and Beclin 1 acetylation by use of salicylic acid. We found that salicylic acid induced the conversion of LC3 I to LC3 II and downregulation of p62 in HCT 116 and SW480 cells. It suggested that salicylic acid induced autophagy and autophagic degradation was not blocked in CRC cells (Supplementary Figure 1A, 1B). We also investigated whether salicylic acid affects the acetylation of Beclin 1 just as aspirin did. But salicylic acid had no effect on Beclin 1 acetylation, either endogenous or exogenous (Supplementary Figure 1C, 1D). In addition, combination with C646 could restore the anticancer effect of aspirin in CRC cells (Figure 5D-5G). These findings indicate that acetylation effect of aspirin rather than function of salicylate group blocks the autophagic degradation and impairs the anticancer effect of aspirin.

Autophagy is thought to play dual roles in cancer [41]. And autophagy is a dynamic and multi-step process. Every step in autophagy flux can be mediated by multiple signaling pathway. Therefore, the study of the detailed molecular mechanisms underlying autophagy is crucial for cancer control. Many studies have shown that autophagy can contribute to apoptosis [42, 43]. In our study, we found that complete autophagic flux enhances aspirin-induced apoptosis, but it also resulted in cell death apoptosis in dependent. These findings suggested that autophagy-mediated cell death also takes part in the antitumor effect of aspirin in CRC cells.

In conclusion, our study provides a new insight into effect of aspirin in colorectal cancer cells. Aspirin-mediated Beclin1 acetylation inhibits autophagosome maturation and degradation, suggesting that the initiation-inducing and degradation-blocking functions of aspirin are worth further exploration for cancer therapy.

## MATERIALS AND METHODS

### Cell culture

The colorectal cancer cell line HCT116 and SW480 were obtained from Cell Lines Bank, Chinese Academy of Science (Shanghai, China). HCT116 cells were cultured in McCoy’s 5A and SW480 cells were cultured in DMEM medium, supplemented with 10% fetal bovine serum (Gibco, Grand Island, NY, USA), 100 mg/mL penicillin and 100 mg/mL streptomycin. The cells were incubated at 37°C in a humidified atmosphere containing 5% CO2.

### Antibodies and reagents

Anti-Beclin 1, acetylated lysine, PARP, p-AMPKα (T172), p-ACC (S79) and p-S6K1 (T389) antibodies were obtained from Cell Signaling Technology (Danvers, MA, USA). Anti-LC3 antibody was obtained from NOVUS (Littleton, CO, USA). Anti-Rubicon antibody was obtained from Abcam (Cambridge, MA, USA). Anti-p300, p62/SQSTM1 and GAPDH antibodies were obtained from Santa Cruz Biotechnology (Santa Cruz, CA, USA). Anti-Flag antibody, aspirin, salicylic acid, Bafilomycin A1 and C646 were purchased from Sigma Aldrich (St. Louis, MO, USA).

### Western blotting and immunoprecipitation

Whole-cell extracts were generated by direct lysis with 1×Cell Lysis Buffer (Cell Signaling Technology) adding 1mM phenylmethylsulphonyl fluoride (PMSF) immediately before use. Samples were boiled by addition 5×SDS sample buffer for 10 min at 100°C and resolved using SDS-PAGE. For immunoprecipitation, cells were lysed by E1A lysis buffer (250mM NaCl, 50mM HEPES (pH 7.5), 0.1% NP-40, 5mM EDTA, protease inhibitor cocktail (Roche)). For acetylation immunoprecipitation, 2 mM TSA and 10mM NAM were added. Cell lysates were mixed with FLAG beads at 4°C overnight. Immunoprecipitates were washed three times with cold lysis buffer and eluted with SDS loading buffer by boiling for 10 min. The proteins were resolved by SDS-PAGE and then transferred onto PVDF membranes, which were blocked in 5% (w/v) nonfat milk and hybridized with specific primary antibodies. The resulting protein bands were visualized using ECL after hybridization with a secondary antibody.

### Plasmids and transfection

The following Addgene plasmids were used: Addgene plasmid 22,418 (mCherry-EGFP-LC3B) and Addgene plasmid 22,405 (GFP-LC3). Flag-Beclin1 was constructed by cloning Beclin 1 into pcDNA3 vector. Site-directed mutagenesis of flag-Beclin 1 was performed using QuikChange XL (Stratagene). Plasmid transient transfection was performed using Lipofectamine 2,000 according to manufacturer’s instructions.

### RNA interference

Target siRNA was produced by GenePharma (Suzhou, China) and transfected using Lipofectamine RNAiMAX Transfection Reagent (life Technologies) according to the manufacturer’s protocol. Target sequence as follows:

p300 (5’-CAGACAAGTCTTGGCATGGTA-3’),

### Autophagy analysis

Autophagy was measured by quantitation of LC3 puncta using fluorescence microscopy. Cells were plated in a 24-well plate on a coverslip and allowed to attach overnight. Cells were infected with appropriate amounts of plasmids GFP-LC3 or mCherry-EGFP-LC3. After aspirin treatment, cells were fixed with 4% paraformaldehyde for 20 min and rinsed with PBS twice. Cells were mounted and visualized under a confocal microscope (Olympus FV-1,000). Total number of cells on images was determined by nuclei staining with DAPI.

### Transmission electron microscopy

Cells were collected and fixed after different treatment. The ultrathin 50 nm sections were cut by use of an ultramicrotome, stained with 2% (w/v) uranyl acetate and lead citrate, then examined with electron microscope Hitachi 600 (Hitachi, Japan).

### Flow cytometric analysis of apoptosis

Apoptotic rate was detected by flow cytometry with the Annexin V-fluorescein isothiocyanate (FITC) apoptosis detection kit (BD). Briefly, cells were collected after different treatment and the assay were performed according to the manufacturer's instruction. Samples were analyzed immediately using a Cytomics FC500 flow cytometer (Beckman Coulter, Miami, FL, USA).

### Cell viability assay

HCT116 and SW480 cells were seeded in 96-well plates at a density of 4,000 cells per well. They were treated with different concentrations of Aspirin for 3 days, and the cell viability was measured by CCK-8 assays.

### Statistical analyses

Student’s t-test was used to compare the differences between two groups. *P< 0.05 was considered statistically significant and **P< 0.01 as highly significant.

## SUPPLEMENTARY MATERIALS FIGURE


