# Supplementary material for: Beclin 1 acetylation impairs the anticancer effect of aspirin in colorectal cancer cells
Source: Oncotarget. 2017 Aug 19;8(43):74781–90. doi: 10.18632/oncotarget.20367 (PMC5650378; doi:10.18632/oncotarget.20367)
Supplement: Supplementary file 1 [file oncotarget-08-74781-s001.pdf]

Beclin 1 acetylation impairs the anticancer effect of aspirin in colorectal cancer cells

SUPPLEMENTARY MATERIALS

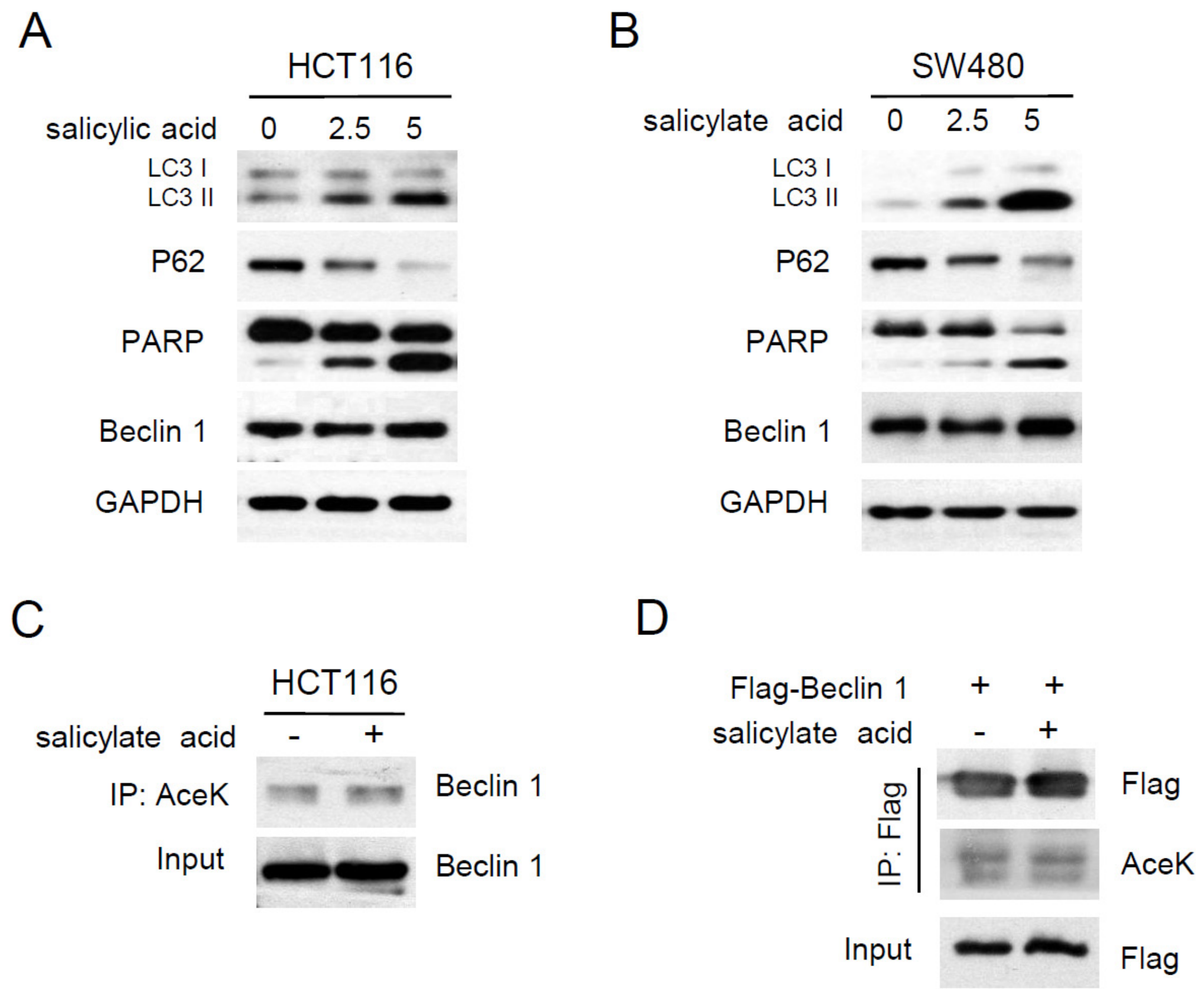

Supplementary Figure 1: Sodium salicylate induced autophagy but not Beclin 1 acetylation in CRC cells. (A-B) HCT116 and SW480 cells were treated with different concentration of sodium salicylate for 24 h. Protein levels were estimated using western blot analysis. GAPDH was used as loading control. **(C)** Acetylated proteins were immunoprecipitated with the antibody to acetylated lysine from HCT116 cells after 5mM sodium salicylate treatment for 24 h. Acetylation of endogenous Beclin 1 protein was analysed with western bolt. **(D)** Flag-tagged Beclin 1 was transfected into HCT116 cells. Then the transfected cells were treated with 5mM aspirin for 24 h or not. Acetylation of exogenous Beclin 1 protein was analysed with IP and western bolt.
